# Metabolic shift towards increased biohydrogen production during dark fermentation in the anaerobic fungus *Neocallimastix cameroonii* G341

**DOI:** 10.1186/s13068-022-02193-z

**Published:** 2022-09-19

**Authors:** Marcus Stabel, Karoline Haack, Hannah Lübbert, Meike Greif, Pascal Gorenflo, Habibu Aliyu, Katrin Ochsenreither

**Affiliations:** Process Engineering in Life Sciences 2: Technical Biology, Karlsruhe Institute of Technology, 76131 Karlsruhe, Germany

**Keywords:** Hydrogen, *Neocallimastigomycota*, Lignocellulose, Metabolites, Biofuel, Biomass usage

## Abstract

**Background:**

Anaerobic fungi of the phylum *Neocallimastigomycota* have a high biotechnological potential due to their robust lignocellulose degrading capabilities and the production of several valuable metabolites like hydrogen, acetate, formate, lactate, and ethanol. The metabolism of these fungi, however, remains poorly understood due to limitations of the current cultivation strategies in still-standing bottles, thereby restricting the comprehensive evaluation of cultivation conditions.

**Results:**

We describe the analysis of growth conditions and their influence on the metabolism of the previously isolated fungus *Neocallimastix cameroonii* G341. We established a bioreactor process in a stirred tank, enabling cultivation under defined conditions. The optimal growth temperature for the fungus was between 38.5 °C and 41.5 °C, while the optimal pH was 6.6–6.8. Like other dark fermentation systems, hydrogen production is dependent on the hydrogen partial pressure and pH. Shaking the bottles or stirring the fermenters led to an increase in hydrogen and a decrease in lactate and ethanol production. Regulation of the pH to 6.8 in the fermenter nearly doubled the amount of produced hydrogen.

**Conclusions:**

Novel insights into the metabolism of *Neocallimastix cameroonii* were gained, with hydrogen being the preferred way of electron disposal over lactate and ethanol. In addition, our study highlights the potential application of the fungus for hydrogen production from un-pretreated biomass. Finally, we established the first cultivation of an anaerobic fungus in a stirred tank reactor system.

**Supplementary Information:**

The online version contains supplementary material available at 10.1186/s13068-022-02193-z.

## Introduction

As consequences of global warming and climate change become increasingly apparent, establishing a circular bio-based economy seems inevitable. Hydrogen is considered a possible green energy carrier in such a bio-based economy and is already highly demanded in different industries today [1, 2]. Up to date, hydrogen is mostly produced from fossil fuels. Lignocellulose containing waste represents a cheap and widely available alternative raw material for hydrogen production. Yet, the recalcitrance of lignocellulose against degradation hinders its efficient deployment for energy generation. The currently available pathways for thermochemical lignocellulose conversion to hydrogen require a high energy investment [3]. Biological processes, by contrast, only require relatively low temperatures and pressures, thereby reducing energy costs. The absence of the requirement for catalyst regeneration also confers an added advantage on biological processes. The known biohydrogen production processes include biophotolysis, photofermentation, bioelectrolysis, the biological water-shift reaction and dark fermentation, which have been reviewed recently [3]. Dark fermentation, peculiarly, enables the conversion of residual biomass to hydrogen through microorganisms without the need for an external light or electricity source. Microbial biomass degradation requires extensive pre-treatment to break down the lignocellulose into fermentable sugars. Pretreatment, however, comes with several drawbacks, including high cost, high energy input, environmental hazards, and the production of fermentation-inhibiting compounds [4]. Anaerobic fungi of the phylum *Neocallimastigomycota* are capable of growing on un-pretreated biomass [5]. These fungi, which originate from the guts of ruminants [6], synthesize a retinue of carbohydrate-degrading enzymes [7].

During growth, mainly hydrogen, acetate, formate, lactate, and ethanol are produced in a bacterial-like mixed acid fermentation [8, 9]. Interestingly, anaerobic fungi harbor both pyruvate ferredoxin oxidoreductase (PFO) and pyruvate formate lyase (PFL) [9], suggesting multiple pathways for hydrogen formation. Central to their metabolism are the hydrogenosomes harbored by the *Neocallimastigomycota* instead of mitochondria. These organelles are thought to have evolved from mitochondria, but do not contain an organelle genome [10, 11]. A detailed insight into anaerobic fungal hydrogenosomes is given by [12].

Despite their biotechnological potential, the knowledge of the culture conditions of anaerobic fungi is limited [13]. Presently, experiments with pure cultures of *Neocallimastigomycota* involve cultivation in still-standing, airtight bottles. Recent studies on basic growth parameters like pH, the influence of agitation, or product inhibition on growth are largely missing, and stirred tank reactors have not been used to the best of our knowledge for studying pure fungal cultures. The application of anaerobic fungi in dark fermentation was reported recently [14]. *Neocallimastix cameroonii* strain G341 has been isolated previously from giraffe feces [15]. Among the isolated strains, it showed the highest carbon source catabolic diversity and the highest robustness. *Neocallimastix* is one of the best-described genera of anaerobic fungi, enhancing the comparison of G341 to other studied strains in a research area where much is unknown. Here, we report the first adaptation of the culture of strain G341 to dark fermentation and its establishment in a small-scale stirred tank reactor, giving novel insights into the fungal metabolism.

## Methods

### Fungal culture and bottle experiments

The used medium, media components, and the fungal strain were described recently [15]. *Neocallimastix cameroonii* strain G341 was maintained by subculture every 6 to 7 days. Therefore, 5 ml of grown culture were inoculated with a syringe into 50 ml of fresh defined medium in a 118-ml serum bottle with 5 g/l wheat straw or cellobiose as carbon source complemented with 0.5 ml antibiotics solution and 0.5 ml vitamin solution. The cultures were incubated still-standing at 39 °C in the dark in a Multitron Shaker (Infors, Bottmingen, Switzerland). These culture conditions were also applied for the experiments described below if not specified otherwise.

#### Determination of temperature optimum

A bottle of defined medium with 5 g/l cellobiose as a carbon source was complemented with vitamin and antibiotic solution. 5 ml of complemented medium was transferred to a 16-ml Hungate tube with a CO_2_ atmosphere through a syringe. Prepared Hungate tubes were inoculated with 0.5 ml of preculture and incubated for 6 days in triplicate at 35 °C, 37 °C, 39 °C, 41 °C, 43 °C, and 45 °C in a BioShake IQ (Analytic Jena). The exact temperature was determined by measurement of an identical setup with water instead of culture in the same shaker as the samples with a submersion thermometer. The pressure was measured daily and hydrogen content was determined at the end of the incubation period.

#### Determination of pH optimum

50 ml of defined medium with a pH of 6.2, 6.4, 6.6, 6.8, or 7.0 was inoculated in triplicate with 1 ml of well-grown culture and incubated at 39 °C in the dark for 6 days. 5 g/l cellobiose was used as sole carbon source. Samples of liquid and gas phases were taken after inoculation and at the end of the experiment. Gas-phase samples were analyzed by GC, while analysis of liquid samples was performed by HPLC.

#### Influence of agitation, carbon source concentration, and headspace volume

50 ml of defined medium was filled either in 250-ml or 118-ml serum bottles and contained 5 g/l or 20 g/l of either cellobiose or straw. After inoculation with 5 ml of culture, the bottles were incubated with shaking (200 rpm) or without (0 rpm) for 4 days at 39 °C in the dark. Each combination was tested in triplicate. The pressure was determined daily. The components of both liquid and gas phases were analyzed, respectively, by HPLC or GC after inoculation and at the end of the experiment.

#### Hydrogen inhibition

Before inoculation 0 ml, 2.5 ml, 5 ml, 7.5 ml, 10 ml, 12.5 ml, 15 ml or 20 ml of hydrogen were added to the headspace of the serum bottle with a syringe at 1 bar. The bottle contained 50 ml of defined medium with 5 g/l cellobiose and was inoculated with 5 ml of preculture. The pressure was measured post-inoculation and after 4 days of incubation and samples from both liquid and gas phases were taken. Samples were analyzed with HPLC or GC.

#### Pressure inhibition

Before inoculation the pressure was adjusted to 1.2 bar, 1.4 bar, 1.6 bar, 1.8 bar, 2.0 bar, 2.2 bar or 2.4 bar by addition of 100% nitrogen through a syringe with a manometer. The bottle contained 50 ml of defined medium with 5 g/l cellobiose. All conditions were inoculated with 5 ml of culture and incubated for 4 days. The pressure was determined on daily basis and samples from the gas phase were analyzed by GC at the beginning and the end of the experiment.

#### Alternative reducing agent and nitrogen sources

Na_2_S was tested as an alternative reducing agent to replace cysteine and was applied in concentrations of 0 mM, 3 mM, and 5 mM. Subsequently, 0.5 mM Na_2_S was used to substitute cysteine when testing alternative nitrogen sources. Here, 5 g/l cellobiose was used as a carbon source. All nitrogen sources were added to the medium post-autoclaving with a final concentration of 19.966 mmol nitrogen from 10 × stock solutions. These were prepared by dissolving the respective nitrogen source in ddH_2_O, adjusting the pH to 6.9 with NaOH/HCl and subsequent filter sterilization. The exact concentrations in g/l for the stock solutions can be found in Additional file 1: Table S1. After inoculation *N. cameroonii* was grown for 5 days before passaging to fresh media, which was repeated twice. In the fourth passage triplicates of each condition were cultivated for 7 days. The pressure was measured on daily basis and samples of the gas phase were taken at the end of the experiment.

### Bioreactor fermentation

A multi-minifermenter system consisting of six 500-ml vessels (“SixFors”, Infors, Bottmingen, Switzerland) was used for bioreactor cultivations. Each bioreactor contained sensors for temperature and pH regulation, sample tubes for liquid and gas phases, a stirrer and a sparger. Tygon^®^-LMT-55 tubes were used for all connections to keep the system airtight. The temperature was regulated through external heating plates and the pH by the addition of anaerobic 4 M H_3_PO_4_ and 1 M NaOH, automatically controlled by the software IRIS 6 (Infors, Bottmingen, Switzerland). Before autoclaving vessels were filled with 300 ml of defined medium containing 20 g/l wheat straw. When 5 g/l cellobiose was used as a carbon source instead of wheat straw, it was added from a 50 g/l stock solution under a sterile hood post-autoclaving. In this case, the volume and the concentration of the other medium components were adapted accordingly. After autoclaving, the medium was complemented by the addition of 3 ml of each vitamin solution and antibiotic solution. The whole system was subsequently anaerobized by sparging with 100% CO_2_ while stirring at 250 rpm. After gassing, 3 ml of sterile cysteine HCl solution (100 g/l) was added to each bioreactor. After one hour the pH was set to 7.1. When this pH value was reached both the gas entry and gas exit of the fermenter were closed, sealing off the fermenter, and the pH was readjusted to 6.8 afterward. Once the parameters were stable the pressure inside the fermenter was adjusted to 1.1 bar with CO_2_ by using a syringe with a manometer. Inoculation was followed with 30 ml of *N. cameroonii* culture grown with 5 g/l cellobiose for 4–5 days. If not specified otherwise all fermentations were performed at 39 °C, pH 6.8, and 250 rpm with a daily sampling of 2 ml liquid phase for HPLC. The pressure was measured with a manometer and 5 ml samples of the headspace were taken with a syringe. Special care was taken to avoid leaching of the gas phase. Sampling was repeated every 24 h. The following parameters were tested in different experiments: rotation velocity of the stirrer (0 rpm, 250 rpm, 600 rpm), turned on vs turned off pH regulation during the fermentation. All reactor experiments were performed in duplicate and the fermentation with cellobiose was performed in quadruplicate.

### Analytics

While sample preparation and measurements were performed as described recently [15], special care was taken during sampling to avoid evaporation of volatile compounds. Therefore, samples were kept on ice at all times or stored at -20 °C when analyses were not performed immediately.

### Statistics

Multiple linear regression analysis was performed with Origin 2019b V.9.65 (OriginLab, Northampton, USA).

## Results

Here we describe the first characterization of the growth conditions of the anaerobic fungus *N. cameroonii* and their effects on metabolite production. Culture pressure correlates with biomass production [16] and increases due to H_2_ and CO_2_ produced by the organism and CO_2_ liberated from the carbonate buffer due to acidification of the media by the produced organic acids. Hence, we used pressure as a fast and qualitative measure for metabolic activity in initial experiments.

### Temperature and pH effects on G341

**Fig. 1:**
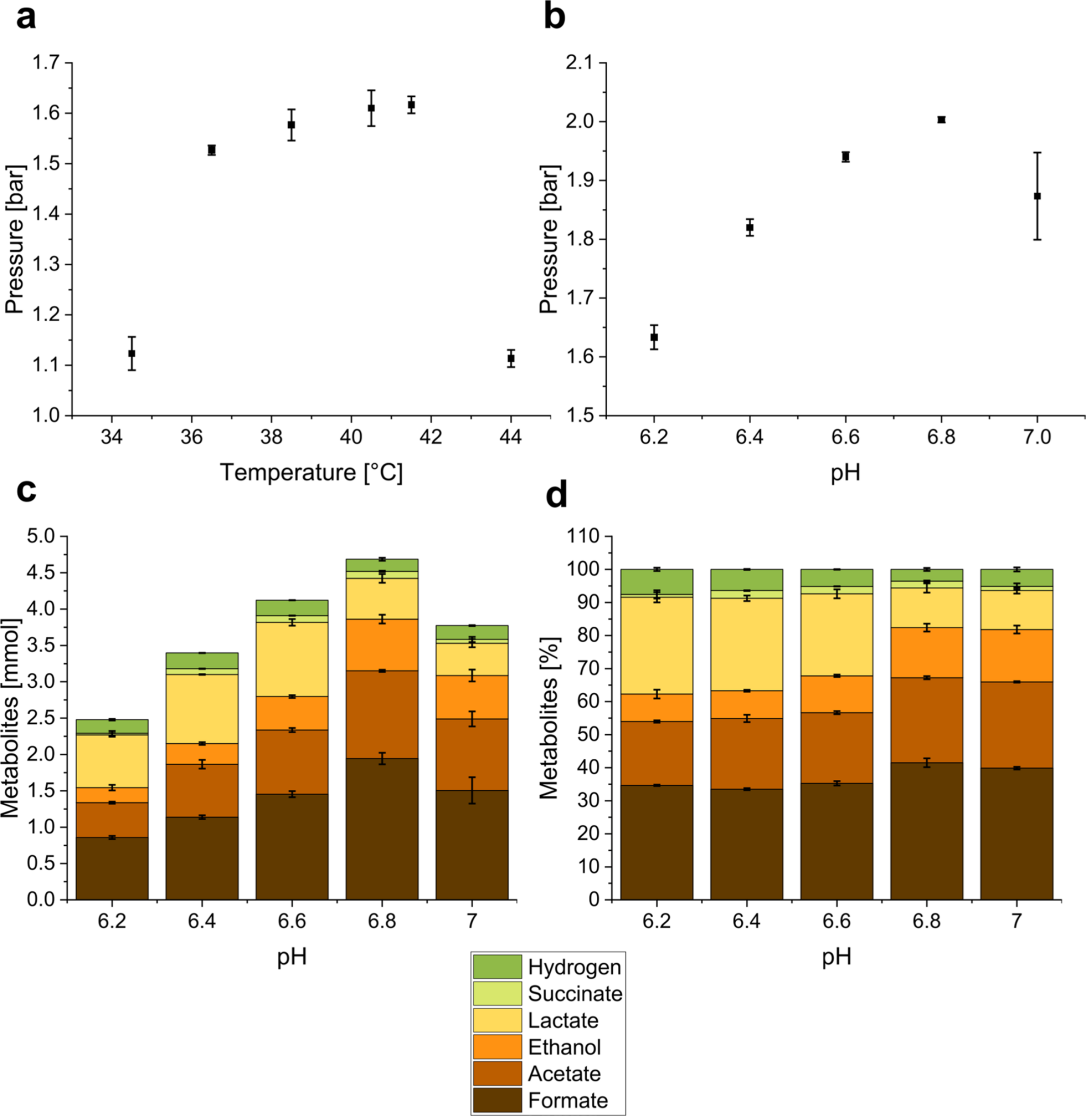
Influence of temperature and pH on growth and metabolite production of N. cameroonii. **a** Influence of the temperature on the pressure during growth; **b** influence of pH on the pressure during growth; **c** influence of pH on the total amount produced metabolites during growth; **d** influence of pH on the relative amounts of produced metabolites compared to the total metabolite amount during growth

To determine the optimal growth temperature, *N. cameroonii* was cultured at temperatures between 34 and 45 °C in Hungate tubes. Analysis of the cumulative pressure revealed a sustained pressure build-up between 36.5 °C and 41.5 °C with a maximum of 1.617 ± 0.017 bar at 41.5 °C (Fig. 1a). Outside this range, the metabolism of the organism was suppressed greatly, as indicated by sharply decreased pressure values of 1.113 ± 0.017 bar at 44 °C and 1.123 ± 0.033 bar at 34.5 °C (Fig. 1a).

Pressure development (Fig. 1b) and metabolite production (Fig. 1c and d) were investigated for initial pH values between 6.2 and 7.0. The highest final pressure was observed at an initial pH of 6.8 (2.003 ± 0.005 bar) and the lowest at pH 6.2 (1.633 ± 0.021 bar) (Fig. 1b), a trend which also reflects the observed tendency in carbon source consumption. The carbon source, cellobiose, was completely hydrolyzed to glucose monomers under all conditions. However, at initial pH values of 6.6 and 6.8, the carbon source was depleted as no residual glucose was detectable, whereas some glucose remained unused at pH 6.2 (0.537 ± 0.020 mmol), 6.4 (0.192 ± 0.011 mmol), and 7.0 (0.067 ± 0.048 mmol). Similarly, the highest maximum metabolite production (4.685 ± 0.048 mmol) was achieved at an initial pH of 6.8 (Fig. 1c). Due to the correlation between pressure increase (Fig. 1b) and total metabolite production (Fig. 1c), and the focus of this study on fungal metabolism, the metabolites form the basis for subsequent inferences.

Additionally, initial pH also affected the total amounts (Fig. 1c) and ratios (Fig. 1d) of the produced metabolites produced. The highest cumulative hydrogen level (0.218 ± 0.002 mmol) was observed in cultures growing at pH 6.4, followed by pH 6.6 (0.212 ± 0.002 mmol). At pH 6.8, the highest amounts of formate (1.945 ± 0.079 mmol), acetate (1.207 ± 0.015 mmol), ethanol (0.711 ± 0.060 mmol), and succinate (0.097 ± 0.009 mmol) were observed but the lowest hydrogen production (0.166 ± 0.020 mmol). The highest lactate production occurred at an initial pH of 6.6 (1.022 ± 0.045 mmol) and of pH 6.4 (0.950 ± 0.004 mmol). When comparing the ratio of the single metabolites to the total amount of metabolites (Fig. 1d) produced, there was a shift from acetate, formate, and ethanol at higher initial pH (6.8) toward lactate and hydrogen at lower pH (6.2–6.6). However, at an initial pH of 7, the relative hydrogen values were elevated compared to pH 6.8.

### Volumetric and bottle agitation effects on metabolite production

**Fig. 2:**
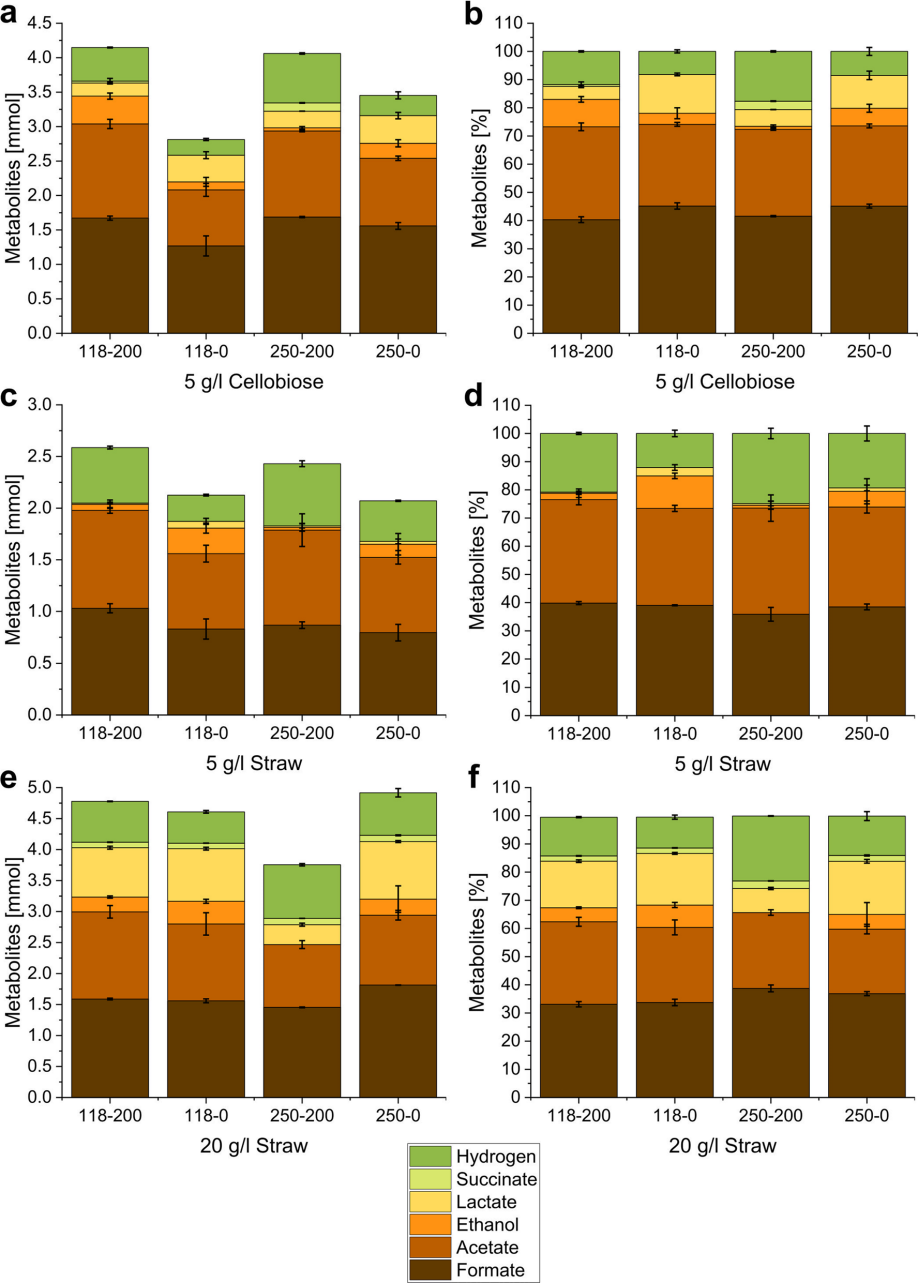
Effect of the bottle volume and agitation on metabolite production during growth on different carbon sources. **a**, **c** and **e** Show the absolute metabolite amounts produced, and **b**, **d**, and **f** are the relative amount of each metabolite to the total amount of produced metabolites. The samples are named after the bottle volume in ml (118 vs 250) and the agitation in rpm (0 vs 200). Please mind the differences in the y-axis between the different carbon sources

To evaluate the influence of culture agitation on metabolic activities, *N. cameroonii* was cultivated in 5 g/L and 20 g/L of either wheat straw or cellobiose combined with agitation at 0 or 200 rpm. Gas-to-liquid ratio effects on net anaerobic metabolism was studied by comparing produced metabolites in cultures cultivated in 118- and 250-ml bottles. The results indicated that no fungal growth occurred in 20 g/L cellobiose culture under all conditions. By contrast, varying metabolic patterns were observed in cultures grown in 5 g/L cellobiose and straw amounts. However, bottle agitation changed culture morphology from mat aggregates formed under still-standing cultivation to pellet-like aggregates in agitated cellobiose cultures (Additional file 1: Fig S1). For straw cultures, no aggregation was observed during agitation. Overall, higher H_2_ yields were observed under agitation and/or in larger bottles compared to the still-standing and/or smaller culture bottles (Fig. 2). However, the effect of bottle volume was smaller than the effect of agitation. The highest amounts of hydrogen were always observed when both conditions were applied, i.e., in agitated 250-ml bottles.

The increase in hydrogen production was accompanied by a decrease in lactate and, in most cases, also a decrease in ethanol amount (Fig. 2). For 250-ml bottles with 5 g/l (Fig. 2c, d) or 20 g/l straw (Fig. 2e, f) the ethanol values were low, resulting in no observable change to net production. Only when using 5 g/l cellobiose in 118 ml bottles more ethanol was produced with agitation than without agitation (Fig. 2a, 2b), leading to the highest total ethanol amounts (0.404 ± 0.045 mmol) of the whole experiment. Furthermore, it represents the only exception to the observed inverse relation between hydrogen and ethanol production. Lactate was detected only in low amounts (0.011 ± 0.009 mmol–0.065 ± 0.029 mmol) in cultures with 5 g/l straw, in comparison to the other conditions and highest with 20 g/l straw (0.322 ± 0.024 mmol–0.929 ± 0.016 mmol). Citrate was only detected in traces in samples of 20 g/l straw cultures. Succinate was produced in the lowest amounts and only in the presence of citrate in agitated culture with cellobiose (0.000 mmol – 0.120 ± 0.004 mmol) and 20 g/l straw (0.089 ± 0.001 mmol–0.101 ± 0.008 mmol). In the latter, succinate values seemed to be uninfluenced by agitation but were slightly higher in 250 ml serum bottles. Both formate and acetate production behaviors varied depending on the carbon source. Like hydrogen, the amounts of the two metabolites increased with agitation and higher gas-phase volume during the growth on cellobiose. In 5 g/l straw cultures, acetate and formate increased with agitation. By contrast, formate amounts seemed to decrease slightly with the higher headspace volume. In 20 g/l straw cultures, acetate amounts were higher in the smaller bottles, but no conclusions could be drawn for formate production. Some conditions, like 20 g/l straw in 250 ml bottle with agitation, showed high deviations of the total amount of produced metabolites when compared to the conditions of the same amount of carbon source, suggesting differences in the growth state.

To verify whether an increase in headspace volume is causative of higher hydrogen production, for instance, due to dilution effects or the reduction of hydrogen partial pressure, experiments were conducted with several different initial hydrogen concentrations. The increase in the initial hydrogen concentration led to an increase in both carbon source consumption (Additional file 1: Tab. S2) and total metabolite production (Additional file 1: Fig. S2a) except for hydrogen itself (Additional file 1: Fig. S2a, Fig. S3a).

When looking at the relative metabolite distribution (Additional file 1: Fig. S2b), an increase in lactate and ethanol and a decrease in formate production could be observed with increasing initial hydrogen concentration.

As the addition of hydrogen also increases total pressure, the influence of elevated initial pressure on hydrogen production was investigated by the addition of nitrogen. Although a pressure increase decreased the hydrogen production (Additional file 1: Fig. S3b), the effect was, however, significantly smaller than the corresponding hydrogen partial pressure as revealed by multiple linear regression with p < 0.05 (Additional file 1: Tab. S3). While raising the pressure (bar) had a coefficient of -0.053 in the analysis, increasing the corresponding initial hydrogen amount (mmol) had a coefficient of -0.142. On addition of hydrogen to the bottle, the maximal difference of initial bottle pressure was 0.226 bar (Additional file 1: Tab S2), suggesting that the reduction of the produced hydrogen by pressure should not surpass 0.012 mmol. The maximal hydrogen production reduction by hydrogen addition was 0.109 mmol, which exceeds the above estimate.

### Alternative reducing agents and nitrogen sources

To evaluate alternative nitrogen sources, the standard reducing agent cysteine, which might also be a usable nitrogen source, had to be replaced by another non-nitrogen-containing reducing agent, for example, Na_2_S. However, in literature, contradicting results on Na_2_S being usable as a reducing agent for anaerobic fungal growth have been reported. While [17] confirmed its suitability, other groups found it to be toxic [9]. In the presented study, culture supplemented with both 0.3 mM and 0.5 mM Na_2_S showed growth. Omitting the reducing agent led to inconsistent growth patterns among the replicates. Applying Na_2_S as a reducing agent, the effects of different nitrogen sources were tested for sustaining the growth of *N. cameroonii* using pressure and produced hydrogen as growth indicators. The fungus grew on glutamine, ammonium sulfate, and ammonium nitrate but not on urea, sodium nitrate, arginine, cysteine, glycine, and straw. When comparing Na_2_S to cysteine as a reducing agent, the variation in growth seemed to be higher (Additional file 1: Tab. S4). As cysteine is also a reducing agent, its toxicity was also tested. *N. cameroonii* grew up to 2 g/l of cysteine, but no growth was detectable at 3 g/l.

### Stirring enhances fermentative hydrogen production

**Fig. 3:**
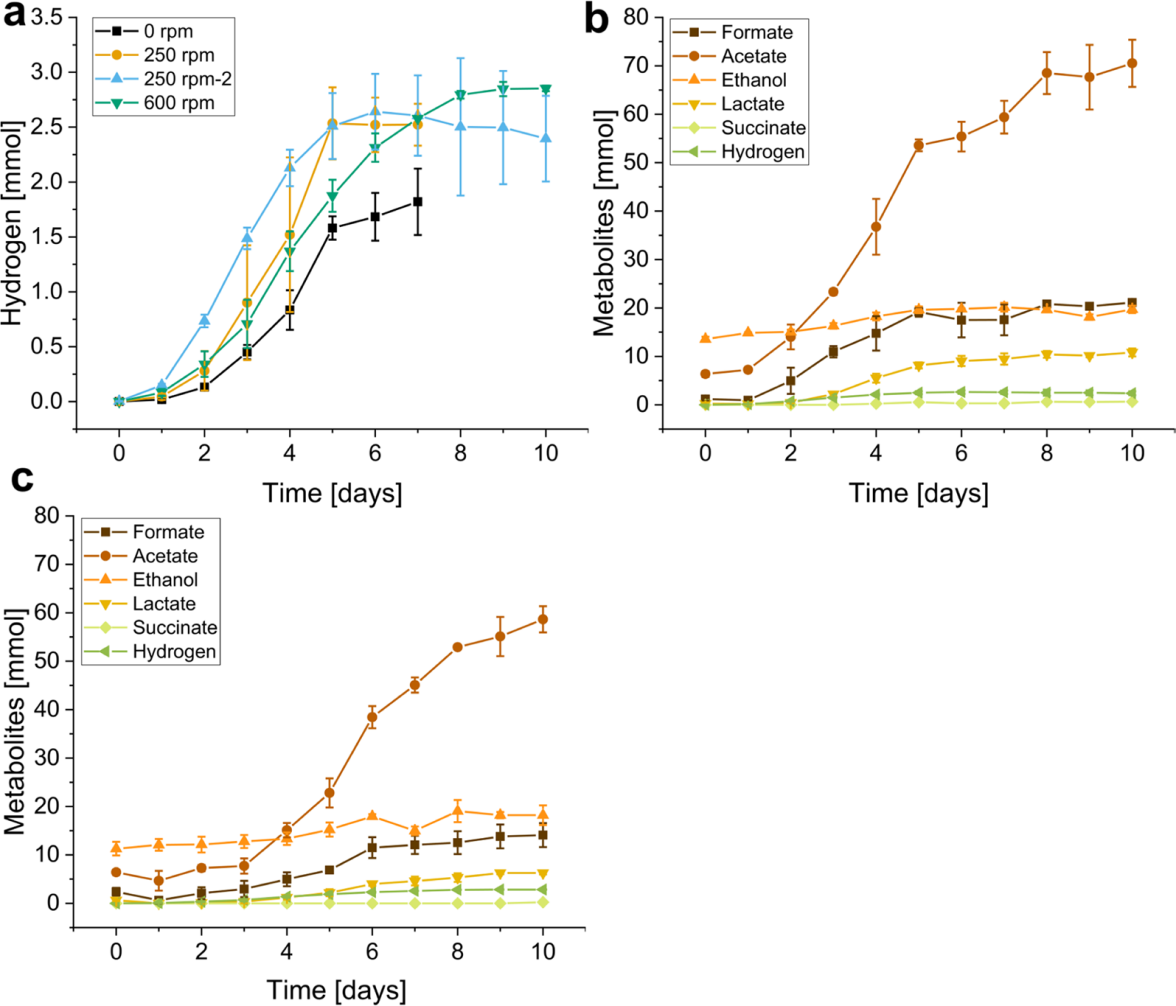
Effect of stirrer velocity on the metabolite production of N. cameroonii. The fungus was grown in a stirred tank reactor with straw as the sole carbon source. **a** Hydrogen evolution during two different setups comparing 0 rpm (black) vs 250 rpm (yellow) and 250 rpm (blue) vs 600 rpm (green). **b** Metabolite production during the second setup with 250 rpm. **c** Metabolite production during the second setup with 600 rpm

Following up on the above agitation experiments in bottles, the effect of stirring on the growth of *N. cameroonii* with straw was investigated in a bioreactor. Figure 3a shows the amount of hydrogen in the headspace for two fermentation experiments. In the first setup, differences between no-stirring and stirring at 250 rpm were investigated, while in the second setup, 250 rpm and 600 rpm were compared. The cultivation length was defined by the duration of hydrogen production, i.e., 6 days and 10 days for the first and second setup, respectively. Hydrogen production was lowest without stirring (1.821 ± 0.302 mmol after 7 days). While stirring at 250 rpm led to the fastest hydrogen production with 2.641 ± 0.345 mmol after 6 days, it was surpassed on day 8 when stirring at 600 rpm (2.795 ± 0.034 mmol). When comparing the other produced metabolites in the second setup (Figs. 3b, c), at 250 rpm (Fig. 3b), 17.497 ± 3.570 mmol of formate was present after the first 6 days. This amount increased further to 21.125 ± 0.419 mmol at the end of the fermentation. By contrast, 9.056 ± 1.043 mmol lactate and 20.164 ± 0.919 mmol ethanol were detected after the first 6 days. The respective amounts changed, only marginally, to 10.835 ± 0.821 mmol and 19.728 ± 0.762 mmol afterward. Acetate amounts continued to increase from 55.386 ± 3.065 mmol to 70.519 ± 4.870 mmol independent of hydrogen production. Succinate could only be detected in trace amounts (0.642 ± 0.049 mmol at day 10) with no deductible relationship with hydrogen. While at 250 rpm the metabolite production seemed to have reached a stationary phase after the 10 days of fermentation, at 600 rpm (Fig. 3c), the metabolites continued to accumulate until the last day with 2.852 ± 0.026 mmol hydrogen, 58.650 ± 2.701 mmol acetate, 14.100 ± 2.480 mmol formate, 18.202 ± 0.302 mmol ethanol, 6.280 ± 0.302 mmol lactate and 0.232 ± 0.232 succinate. This suggests an overall slower growth of the fungus at higher stirrer velocities. An overview of the produced amounts of each metabolite is presented in Table 1.

**Table 1:** Dependence of metabolite production from stirring velocity

		ΔHydrogen [mmol]	ΔAcetate [mmol]	ΔFormate [mmol]	ΔEthanol [mmol]	ΔLactate [mmol]	ΔSuccinate [mmol]	Total [mmol]
250 rpm	Ø	2.392	64.154	19.918	6.164	10.589	0.642	103.859
	SD	0.393	5.438	0.820	0.249	1.067	0.049	2.860
600 rpm	Ø	2.852	52.231	11.706	6.913	5.641	0.232	79.575
	SD	0.026	3.243	3.283	3.440	0.136	0.232	3.157

Metabolites are produced during stirred tank reactor fermentation of N. cameroonii G341 with straw as the sole carbon source depending on the stirrer velocities of 250 rpm and 600 rpm. Displayed are the final amount after 6 days (250 rpm) and 10 days (600 rpm) of cultivation. Ø is the mean value of the duplicate and SD the corresponding standard deviation

### pH regulation improves metabolite production

**Fig. 4:**
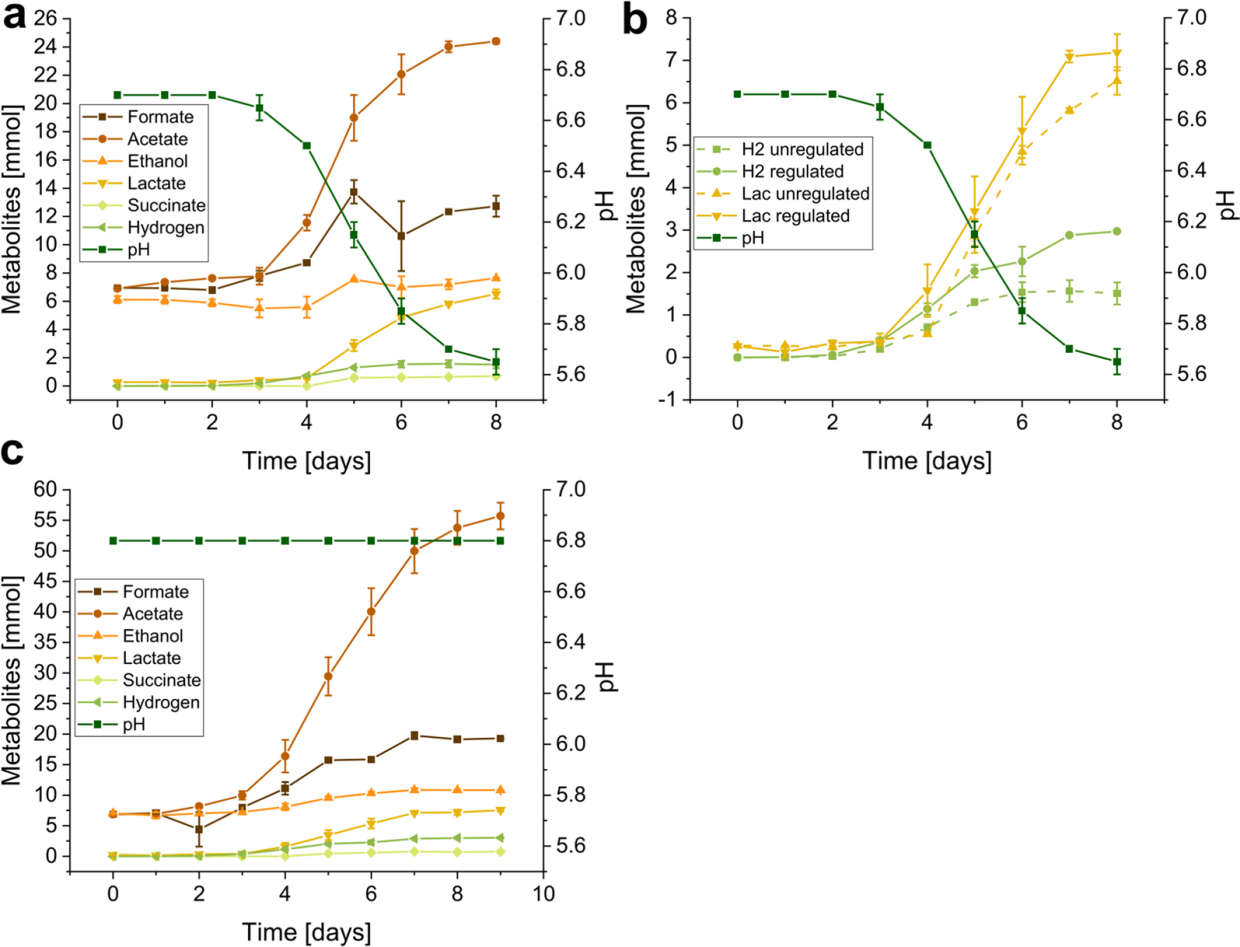
Effect of pH regulation on the metabolite production of N. cameroonii G341. The fungus was grown in a stirred tank reactor with straw as the sole carbon source. **a** Metabolite production and pH development during growth without pH regulation; **b** comparison of pH regulated and unregulated lactate and hydrogen production. pH from the unregulated fermenters is shown and pH for the regulated was kept constantly at 6.8; **c** metabolite production and pH development during growth with pH regulation

To determine the effects of pH regulation on the growth and metabolite production of *N. cameroonii,* a fermentation omitting pH regulation was performed and compared to fermentation with constant optimal pH. In contrast to cultures with pH regulation, the cultivation medium with no pH control progressively becomes acidic, with the pH decreasing gradually from pH 6.8 to 5.65 ± 0.05 on day 8 (Fig. 4). In general, the amounts of all produced metabolites were higher with pH regulation than without (Table 2). The production of hydrogen also seemed dependent on the pH. After reaching pH 5.85 ± 0.05, hydrogen increased marginally from 1.539 ± 0.235 mmol to 1.568 ± 0.254 mmol on day 7 and decreased to 1.509 ± 0.259 mmol on day 8 post-inoculation (Fig. 4a). Similar to the second setup of the stirring experiment (250 rpm vs 600 rpm), the amount of formate also increased from 10.612 ± 2.478 mmol to 12.733 ± 0.750 mmol on days 6 and 8, respectively. By contrast, lactate increase continuously independent of hydrogen production and pH starting between days 2 and 3 (Fig. 4b) with a total amount of 6.517 ± 0.325 mmol and 7.189 ± 0.426 mmol under unregulated and regulated conditions, respectively. Likewise, ethanol production without pH control seemed to start around day 4, reaching 7.638 ± 0.021 mmol at the end of the fermentation. With pH regulation (Fig. 4c), ethanol was produced constantly from the start reaching 10.849 ± 0.086 mmol on day 7 and decreased slightly on day 8. By contrast, acetate amounts increased both without and with pH regulation, reaching 24.402 ± 0.167 mmol and 53.765 ± 2.770 mmol, respectively. Succinate was, however, produced in small amounts (0.706 ± 0.051 mmol vs 0.685 ± 0.082 mmol) under both conditions.

**Table 2:** Dependence of metabolite production from pH regulation

		ΔHydrogen [mmol]	ΔAcetate [mmol]	ΔFormate [mmol]	ΔEthanol [mmol]	ΔLactate [mmol]	ΔSuccinate [mmol]	Total [mmol]
reg−	Ø	1.509	17.490	5.785	1.520	6.238	0.706	33.247
	SD	0.259	0.243	0.831	0.227	0.319	0.051	1.411
reg +	Ø	2.972	46.898	12.261	3.841	6.926	0.685	73.583
	SD	0.016	2.662	0.035	0.023	0.454	0.082	3.194

Metabolites produced during pH unregulated (reg-) and pH regulated (reg +) stirred tank reactor fermentation of N. cameroonii. Ø is the mean value of the duplicate and SD the corresponding standard deviation

### Cellobiose as a carbon source

**Fig. 5:**
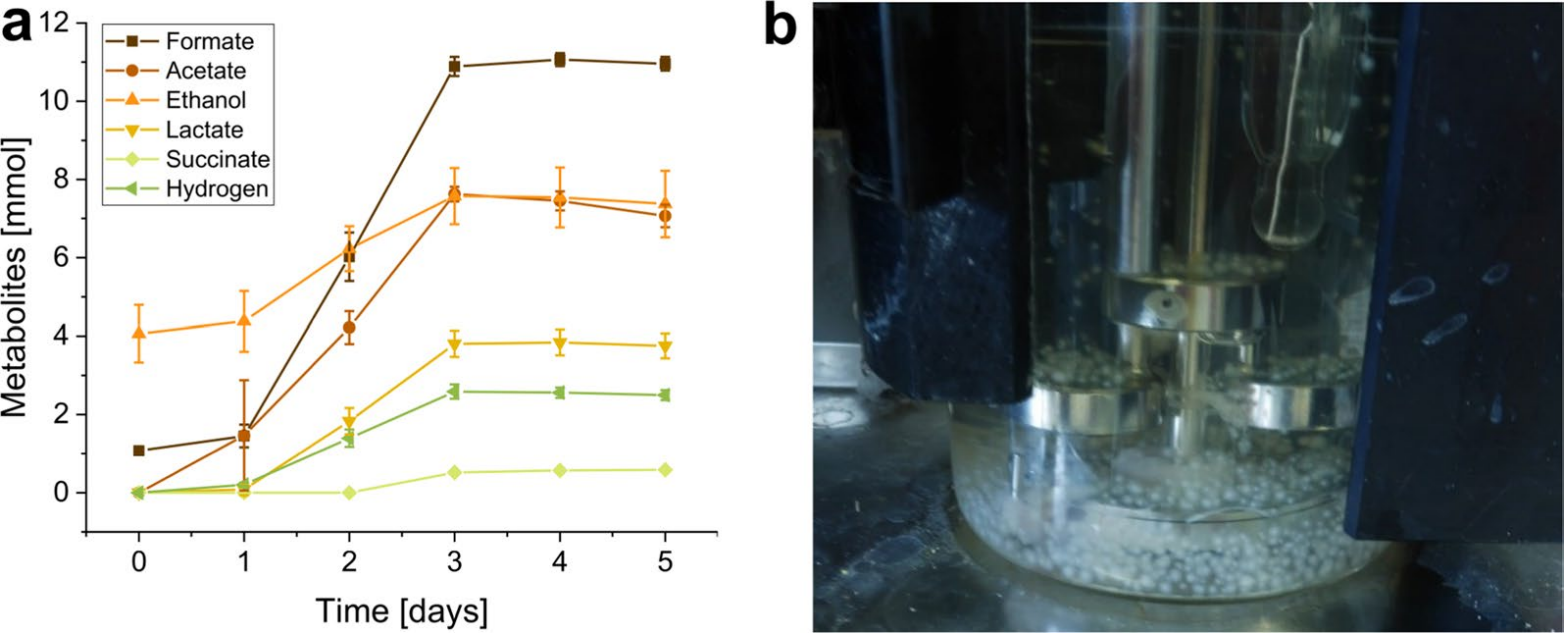
Stirred tank reactor fermentation of N. cameroonii with cellobiose as carbon source. **a** Metabolite production. **b** Fungal pellets in the reactor at the end of fermentation

As the application of straw as a carbon source complicates the calculation of molar yields, bioreactor fermentation with 5 g/l cellobiose as the sole carbon source was performed. Compared to the straw experiments, the overall metabolite production was much faster with cellobiose, but it ceased on day 3 (Fig. 5a). In contrast to the other bioreactor experiments with straw, formate was the metabolite produced in the highest amounts (9.503 ± 0.123 mmol), followed by acetate (7.065 ± 0.288 mmol). Ethanol and lactate production amounted to 3.308 ± 1.156 mmol and 3.754 ± 0.316 mmol, respectively. Succinate was detected in trace amounts (0.585 ± 0.016 mmol). Table 4 shows the calculated yields for each metabolite, and the carbon recovery rate was 0.838 ± 0.052. As observed in the bottle experiments, the fungus formed pellets (Fig. 5b) when grown in cellobiose at 250 rpm in the Sixfors reactors.

### Yields

To compare the performance of bottle and bioreactor experiments, yields for the conditions with the highest hydrogen production for each carbon source were calculated (Tables 3, 4), i.e., bottles with 250 ml volume and grown at 200 rpm vs reactor cultivations with straw and 600 rpm stirring. When using straw as a carbon source, the yield calculations proved difficult because separating the fungal cell mass and residual straw at the end of the fermentation was impossible, preventing the determination of substrate consumption. Therefore, the presented yields assume total consumption and led most likely to underestimation. Nevertheless, when comparing the same carbon sources within the different cultivation setups, the hydrogen yield was higher in bottles, while lactate and ethanol yields were higher in the reactor fermentations. While the acetate and formate yields were similar with cellobiose as a carbon source, growth on 20 g/l straw led to higher formate and much higher acetate yields in the reactor. No reactor experiment using 5 g/l of straw was performed, but in the bottle experiments, the overall metabolites yield, except lactate, was higher when compared to 20 g/l straw.

**Table 3:** Highest yields recorded for each carbon source during bottle experiments

5 g/l cellobiose: yield metabolite/equivalent glucose [mmol/mmol]						
Metabolite	Hydrogen	Acetate	Formate	Ethanol	Lactate	Succinate
Ø	0.491	0.855	1.155	0.031	0.165	0.082
SD	0.007	0.009	0.006	0.012	0.001	0.003
5 g/l straw: yield metabolite/straw [mmol/g]						
Metabolite	Hydrogen	Acetate	Formate	Ethanol	Lactate	Succinate
Ø	2.406	3.679	3.470	0.107	0.058	0.000
SD	0.114	0.633	0.127	0.151	0.082	0.000
20 g/l straw: yield metabolite/straw [mmol/g]						
Metabolite	Hydrogen	Acetate	Formate	Ethanol	Lactate	Succinate
Ø	0.866	1.013	1.454	0.000	0.322	0.100
SD	0.019	0.064	0.007	0.000	0.024	0.000

Ø is the mean value of the duplicate and SD the corresponding standard deviation

**Table 4:** Highest yields recorded for each carbon source during stirred tank reactor experiments

5 g/l cellobiose: yield metabolite/equivalent glucose [mmol/mmol]						
Metabolite	Hydrogen	Acetate	Formate	Ethanol	Lactate	Succinate
Ø	0.285	0.806	1.084	0.377	0.428	0.067
SD	0.014	0.033	0.014	0.132	0.036	0.002
20 g/l straw: yield metabolite/straw [mmol/g]						
Metabolite	Hydrogen	Acetate	Formate	Ethanol	Lactate	Succinate
Ø	0.475	8.705	1.951	1.152	0.940	0.039
SD	0.004	0.541	0.547	0.573	0.023	0.039

Ø is the mean value of the duplicate and SD the corresponding standard deviation

## Discussion

Knowledge of the nutritional needs of *Neocallimastigomycota* fungi is generally scarce [13]. Despite the recent discovery of several novel genera and species over the last years, no efforts have been invested in characterizing the basic needs, like the pH or temperature range of these fungi. Here, we showed the pronounced influence of pH and hydrogen partial pressure on the metabolism and established the cultivation of anaerobic fungus in a stirred tank reactor system for the first time. Finally, the hydrogen yields of other dark fermentation studies are compared, highlighting the potential of anaerobic fungi for hydrogen production.

### Growth conditions influence metabolite production

Our analysis showed that *N. cameroonii* strain G431 grows optimally at temperatures between 38.5 °C and 41.5 °C, conforming to the previous description of the genus *Neocallimastix* [17, 18]. In contrast, the majority of studies dealing with the cultivation of other *Neocallimastigomycota* species were conducted only around 39 °C [13], which is in the optimal range of *N. cameroonii* but slightly below the temperatures (40.5–41.5 °C) under which the highest hydrogen production and pressure were observed from the current study. Our data highlight the temperature sensitivity of *N. cameroonii,* as suggested by the rapid decrease in growth observed outside the optimum temperature range. More studies could help elucidate if the generally used 39 °C represents the true optimal growth temperature for all species of anaerobic fungi, especially with a future biotechnological application of the organisms in mind. Initial pH values of 6.6 and 6.8 are considered optimal, as these were the only conditions with total carbon source consumption. Previous studies reported that other *Neocallimastix* species grow or germinate optimally at pH range 6.5–7.5 [17] or 5.5–6.4 [18], respectively.

At lower pH values, the dynamics switched from the production of acetate, formate, and ethanol to lactate and hydrogen. These dynamics matched the observed metabolite profile from the bioreactor experiments with pH control. While metabolites production was much lower without pH regulation, lactate production was fairly stable. Furthermore, our results revealed a pH modulation of hydrogen production. Both initial pH values of 6.4 and 6.6 in bottles and a constant pH of 6.8 in bioreactors resulted in higher hydrogen amounts. In bioreactors without pH regulation, hydrogen production declined considerably when the pH dropped below 6.6. However, the exact modulation by pH will have to be elucidated in future studies. In contrast to bottle experiments, bioreactor fermentations allow much closer control of parameters like pH and will therefore be helpful in subsequent studies.

### Hydrogen is the main influence on metabolite production

**Fig. 6:**
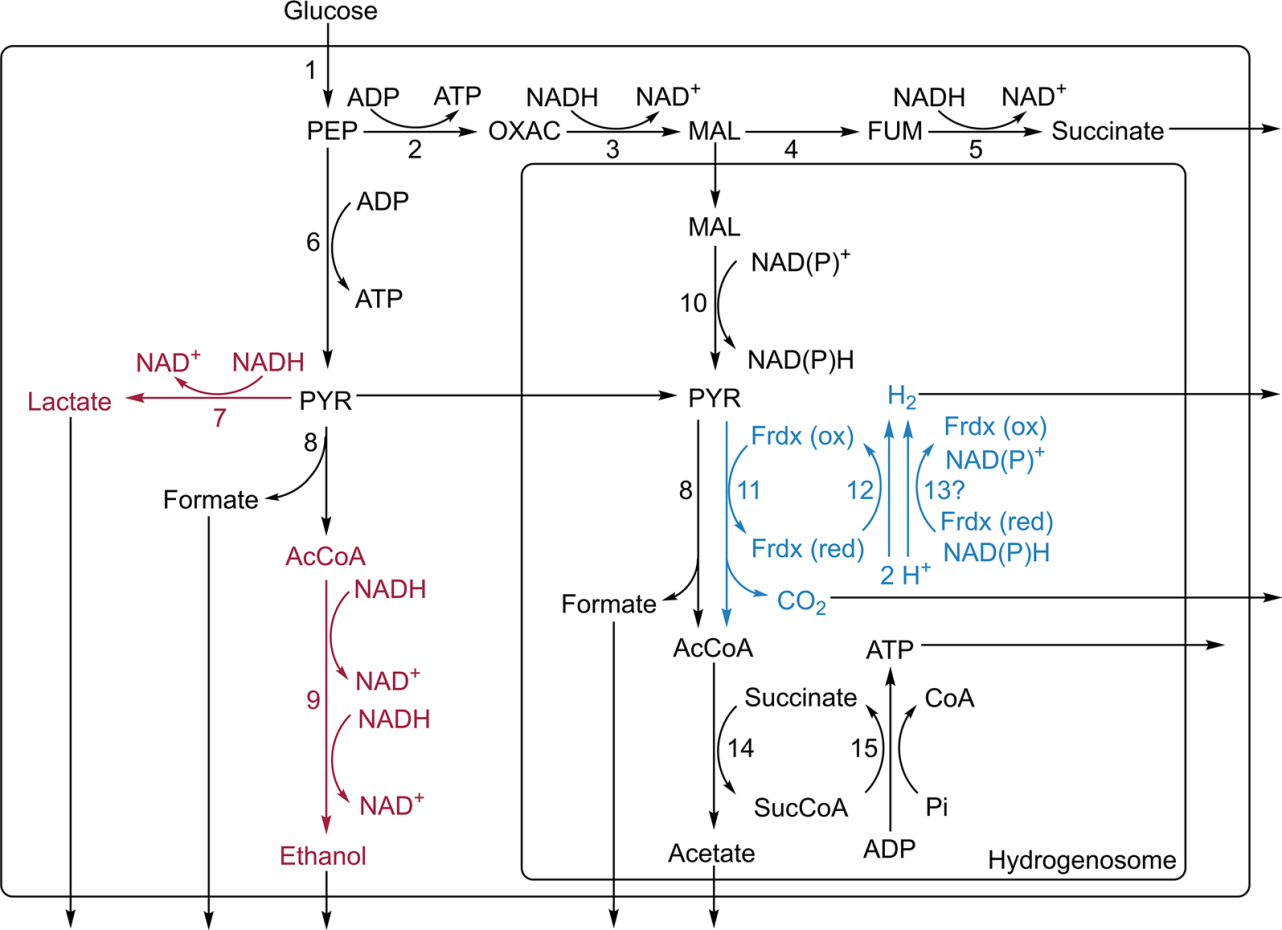
Metabolic pathways from Neocallimastix cameroonii. Adopted from [8, 9]. Primary and preferential electron disposal for hydrogen production is marked blue. Secondary cytosolic electron disposal is marked red. Enzymes are marked as numbers: 1. Embden–Meyerof pathway, 2. phosphoenolpyruvate carboxykinase, 3. malate dehydrogenase, 4. fumarase, 5. fumarate reductase, 6. pyruvate kinase, 7. lactate dehydrogenase, 8. pyruvate formate lyase, 9. alcohol dehydrogenase E, 10. malic enzyme, 11. pyruvate ferredoxin oxidoreductase, 12. ferredoxin hydrogenase, 13?. Possible bifurcating hydrogenase, 14. acetate:succinate CoA transferase, 15. succinyl-CoA synthetase. PEP, phosphoenolpyruvate; OXAC, oxaloacetate; MAL, malate; FUM, fumarate; PYR, pyruvate; AcCoA, acetyl coenzyme A; CoA, coenzyme A; Frdx, ferredoxin; SucCoA, succinyl coenzyme A

To date, anaerobic fungi are cultivated exclusively in still-standing bottles [13]. In this study, we showed that increasing the ratio of gas-phase to liquid-phase and bottle agitation enhanced hydrogen production and, in most parts, also end-product yields in-bottle fermentation. The bioreactor experiments confirmed the above observation, where the hydrogen production increased with stirrer velocity. Previous studies have revealed the influence of partial pressure on dark fermentative hydrogen production [19–21]. High hydrogen partial pressures shift the metabolic flux from hydrogen to other fermentation products. Here, the cultivation of *N. cameroonii* in bottles, with the addition of hydrogen, resulted in a lower amount of produced hydrogen (Fig. 6). However, agitation appears to compensate for the effect of the partial pressure in two ways, as seen by the influence of agitation in the bottle experiments. Firstly, it increases the mass transfer between the liquid and gaseous phase, which decreases the concentration of dissolved and inhibiting hydrogen. Secondly, it prevents the typical mat formation in the fungus. Under still-standing conditions, produced gas can accumulate under the mat, which causes high local hydrogen concentrations in the vicinity of growing fungus, thereby inhibiting the hydrogen production. In the reactor experiments, the applied stirring regime did not disrupt fungal cell mats completely. In this case, only a single Rushton turbine near the vessel bottom was used. For better morphology control and general optimization of the process, several stirrer types should be tested at different heights of the reactor.

### Hydrogenosomal metabolism

Previous studies [8, 9] showed that the amount of produced formate molecules is proportional to the sum of the produced ethanol and acetate molecules. While [8] used *Pyromyces* as organism and fructose as a carbon source, [9] used a related *Neocallimastix* strain and 5 g/l cellobiose as a carbon source. In anaerobic fungi, pyruvate is converted to acetyl-CoA through a pyruvate formate lyase (PFL), thereby producing one molecule of formate for each molecule of acetyl-CoA. PFL is located in both cytosol and hydrogenosome [22]. In further pathways, acetyl-CoA is then converted to ethanol or acetate. In this study, exceptions to the ratio between formate, acetate and ethanol appeared. In bioreactor experiments with straw, the amount of produced acetate alone tripled the amount of formate. However, when the carbon source was 5 g/l cellobiose, under the similar setup, the equimolar ratio was approximated. Likewise, the analysis of most of the bottle experiments showed the equimolar ratio of formate to ethanol and acetate. These results highlight a probable dependency of the metabolite ratio on the culture conditions.

Recently, more insights into the metabolic pathways of anaerobic fungi have been given [9]. Besides PFL, anaerobic fungi also possess a pyruvate ferredoxin oxidoreductase (PFO), located in the hydrogenosome. PFO catalyzes the conversion of pyruvate to acetyl-CoA. The usage of one enzyme over the other carries no energetic cost [9]. While PFL produces formate as a by-product, PFO produces not only CO_2_ but also reduced ferredoxin. Reduced ferredoxin is used to produce hydrogen through a ferredoxin hydrogenase or a bifurcating hydrogenase. So far, only homologous sequences of the latter enzyme have been found in *Neocallimastix* [9]. The advantage of using PFO over PFL would be the disposal of electrons through hydrogen production, with the prerequisite of the presence of a bifurcating hydrogenase. If all electrons were disposed of this way, only hydrogen, formate, and acetate would be metabolic end-products. Thus, alternative options for electron disposal, like the production of ethanol and lactate, would not be necessary. This was observed for the shaken 250-ml bottle with 5 g/l straw as a carbon source, which barely produced lactate and ethanol compared to the total metabolites. By contrast, culture conditions resulting in relatively low hydrogen production, like the still-standing bottles, showed increased lactate and ethanol production. A similar phenomenon has been reported in the co-culture of anaerobic fungi with methanogens. The latter attaches to the fungal rhizoid [23] and consumes produced formate and hydrogen [24]. The consumption of the hydrogenosomal end-products leads to an increase in the hydrogenosomal pathways and a decrease in the cytosolic pathways, like lactate fermentation [25]. In contrast to our findings, however, the above study reported no differences in ethanol production between fungal monoculture and co-culture.

### Secondary electron disposal in *N. cameroonii*

As mentioned above, the increase of hydrogen production during agitation or a by providing larger headspace volume in the bottle experiments resulted in a decrease in lactate and ethanol production. Hydrogen, lactate, and ethanol production are all options to dispose of electrons. The hydrogen-dependent increase/decrease in the lactate and ethanol amounts suggests a shift from hydrogen to lactate and ethanol when hydrogen production becomes energetically unavailable. In the bioreactor experiments, we observed both a dependency and an independency between lactate and hydrogen production. In the stirring experiment, it seemed that the production of all metabolites was stopping at the same time-point as lactate and hydrogen production. This suggests that lactate production ceased because of the lack of fungal growth and not the ceasing of hydrogen production. Conformingly, the lactate production in bioreactor cultivations testing pH regulation continued long after the discontinuation of the hydrogen production. Here, lactate production was similar for both conditions (pH regulated vs unregulated) despite one producing more than twice the amount of total metabolites, suggesting a continuous lactate production, which is independent of the conditions. Unlike other metabolites in the same experiment, we noticed that lactate and ethanol production did not start on the first day. During the agitation bottle experiments, no lactate was produced under some conditions. Previously, it was suggested that the production of anaerobic fungal metabolites is sequential, with lactate, ethanol, and succinate being produced later than hydrogen, formate, and acetate [15]. Future studies will have to elucidate if and how lactate production is regulated. We are inclined to speculate that lactate is used as a secondary electron acceptor in the event of the unavailability of the hydrogen-producing pathways and might be regulated accordingly. Ethanol and succinate, which may function in the electron disposal system, are unlikely to perform this metabolic function in anaerobic fungi. While succinate was only detected in traces, the amount of ethanol was lower than lactate in nearly all experiments. While lactate can be derived directly from pyruvate, ethanol is derived from acetyl-CoA, requiring one additional catalytic step. Electron disposal through ethanol synthesis could compete directly with energy production through acetate synthesis, with acetyl-CoA being the substrate for both processes. This higher catalytic effort combined with the concurrence of electron disposal with energy production could explain the preference for lactate in *N. cameroonii* G341.

### Dark fermentation

With this being the first report establishing anaerobic fungi as organisms for dark fermentation, the procedure still requires further refinement. One limitation is the observed high deviation of produced metabolites in some cases, which may be due to differences in the growth state of the inoculum. A homogenous inoculation is presently unrealizable because the fungus characteristically forms a dense rhizoid. When using other fungi, usage of equal amounts of spores or conidia tends to serve as a method to guarantee homogeneous inoculation. By contrast, anaerobic fungi reproduce via zoospores. A recent study has reported a zoospore harvesting method [26], thereby paving the way for future use of a defined amount of zoospores towards an inoculum standardization for the cultivation of anaerobic fungi.

While the yields for hydrogen were higher in the bottle experiments, the lactate and ethanol yields were generally higher in bioreactor experiments. As described above, increasing the gas-phase/liquid-phase ratio and/or disrupting the mat formation of the fungus increased hydrogen production. In serum bottles, this ratio was higher than in the reactors. In addition, stirring could not provide complete prevention of mat formation. Combined, both effects provide the basis for the observed metabolic shift from hydrogen to lactate and ethanol. Above, we discussed the effect of pH regulation on metabolite production. The highest yield (Table 5) was calculated for the bottle experiments and, therefore, without pH regulation. A higher metabolites yield could be achieved, potentially, by further process optimization in the reactor. Previous studies showed that continuous flow culture increases the substrate consumption by *Neocallimastix hurleyensis* (later reclassified as *Neocallimastix frontalis* [27]) at high substrate concentrations [28]. Here, we also noticed a decrease in yield with the higher substrate concentrations of 20 g/l. An older study reported stimulation of zoospore germination and growth by acetate, iso-butyrate, and 2-methylbutyrate [18]. By contrast, the inhibitory effect of fermentation products, including acetate, on anaerobic fungi growth was also published [29]. While the hydrogen inhibitory effect has been reported, the roles of other produced metabolites and the reason for growth inhibition at higher substrate concentrations remain unresolved.

**Table 5:** Hydrogen production through dark fermentation of wheat straw

Pretreatment	Organism	H_2_ Yield	Publication
Steam acid + enzymatic hydrolysis	*Caldicellulosiruptor saccharolyticus*	3.43 mol/mol sugar	[36]
CaO hydrolysis	Thermal pretreated anaerobic sludge	114 ml/g total solid	[37]
Dilute acid	*Escherichia coli* WDHL	140.1 ml/g total sugars	[38]
Dilute acid + enzymatic hydrolysis	Mixed sludge	141 ml/g volatile solids	[39]
None	*Neocallimastix cameroonii*	2.406 mmol/g = 58.84 ml/g*	This study

Comparison of pre-treatment, used organisms, and hydrogen yields of different studies using dark fermentation of wheat straw. *Conversion of mmol to ml hydrogen following the ideal gas law with temperature 21 °C and pressure 1 bar

Table 5 gives an overview of the hydrogen yields from the wheat straw in different studies and compares the used pre-treatment methods. A comparison between the various studies proved hard due to the differences in pre-treatment and the application of dissimilar pre-treatment fractions in the subsequent fermentation. While other studies used extensive pre-treatment, breaking down complex biomass-derived substrates to their sugar monomers, in this study, we only milled straw into smaller particles. Contrary to previous studies, which reported yields based on the total solids, volatile solids, or sugar content of the straw, our yield estimates are derived directly from the mass of the applied straw. Nevertheless, we obtained about a half to one-third of the yield reported in other studies , further suggesting the robustness of the strategy reported in the current work. Apart from optimizations of fungal growth conditions, this yield could be further increased by follow-up processes using the acids produced by the fungus for additional hydrogen production. The potential integration of anaerobic fungal dark fermentation with photofermentation and biological electrolysis has been discussed recently [14]. Photofermentation is a process of purple non-sulfur bacteria that can gain energy through light but require a carbon source consisting of organic acids like acetate, lactate, or succinate [30]. Hydrogen is generated through a nitrogenase and serves as an outlet for excessive electrons in the absence of nitrogen. In bioelectrolysis, bacteria attached to the anode of electrolysis cells produce CO_2_, electrons, and protons by oxidizing organic acid [31, 32]. The anode serves as the electron acceptor, and the electrons get transferred to the cathode via an external circuit. At the cathode, the protons react with the electrons to form hydrogen. The endothermic barrier to these reactions is overcome by supplying an external voltage [32].

While *N. cameroonii* contains PFL, it lacks a formate hydrogen lyase complex (FHL) present in *Escherichia coli*. FHL catalyzes the reaction of formate to hydrogen. The deletion of an FHL repressor led to increased hydrogen production in the bacterium [33]. The introduction of such an enzyme into *N. cameroonii* could potentially increase the hydrogen yield. A similar effect may be achievable by deleting the PFL pathway to redirect the metabolic flux towards PFO. So far, the genetic manipulation of anaerobic fungi remains in its infancy, with the best result leading to a transient expression of a β-glucuronidase after biolistic transformation [34]. The availability of such a technology could further enhance the research of these organisms and open the doors for biotechnological applications of these remarkable biomass degraders. While methods of permanent transformation are not available, a recent study reported the use of RNA interference to knock down the lactate dehydrogenase of the anaerobic fungus *Pecoramyces ruminantium* [35].

## Supplementary Information

**Additional file 1: Table S1.** Stock concentrations and final concentrations of different N-sources during the testing of these. **Table S2.** Residual glucose and produced hydrogen after growth of N. cameroonii in dependence from the amount of hydrogen added pre inoculation and the pressure inside the bottle at the beginning of the experiment. All values are mean values of a triplicate with standard deviation. **Table S3.** Output from the multiple linear regression in Origin evaluating the effects of the parameters hydrogen start (mmol) and pressure start (bar) on the dependent variable produced hydrogen (mmol). **Table S4.** Final pressure and produced hydrogen during growth on different N-sources with 0.5 mM Na2S as reducing agent. **Figure S1.** Morphology of Neocallimastix cameroonii during growth on cellobiose while agitated. **Figure S2.** Effect of hydrogen addition on the metabolism of N. cameroonii. a: absolute amounts of the produced metabolites; b: relative amount of the produced metabolites in relation to the total amount of produced metabolites. **Figure S3.** Effect of initial hydrogen and pressure on hydrogen production of N. cameroonii. a: Effect of initial hydrogen; b: effect of initial pressure. Linear fitting was performed with Origin (OriginLabs)
