# Supplementary material for: Metabolic shift towards increased biohydrogen production during dark fermentation in the anaerobic fungus Neocallimastix cameroonii G341
Source: Biotechnol Biofuels Bioprod. 2022 Sep 19;15:96. doi: 10.1186/s13068-022-02193-z (PMC9484062; doi:10.1186/s13068-022-02193-z)
Supplement: Supplementary file 1 — Additional file 1: Table S1. Stock concentrations and final concentrations of different N-sources during the testing of these. Table S2. Residual glucose and produced hydrogen after growth of N. cameroonii in dependence from the amount of hydrogen added pre inoculation and the pressure inside the bottle at the beginning of the experiment. All values are mean values of a triplicate with standard deviation. Table S3. Output from the multiple linear regression in Origin evaluating the effects of the parameters hydrogen start (mmol) and pressure start (bar) on the dependent variable produced hydrogen (mmol). Table S4. Final pressure and produced hydrogen during growth on different N-sources with 0.5 mM Na2S as reducing agent. Figure S1. Morphology of Neocallimastix cameroonii during growth on cellobiose while agitated. Figure S2. Effect of hydrogen addition on the metabolism of N. cameroonii. a: absolute amounts of the produced metabolites; b: relative amount of the produced metabolites in relation to the total amount of produced metabolites. Figure S3. Effect of initial hydrogen and pressure on hydrogen production of N. cameroonii. a: Effect of initial hydrogen; b: effect of initial pressure. Linear fitting was performed with Origin (OriginLabs) [file 13068_2022_2193_MOESM1_ESM.docx]

# Tables

Table 1: Stock concentrations and final concentrations of different N-sources during the testing of these.

| N-source | MW [g/mol] | N-Atoms/Molecule | Final concentration [g/l] | Stock 10x [g/l] |
| --- | --- | --- | --- | --- |
| Glutamine | 146.15 | 2 | 1.459 | 14.590 |
| Ammonium sulfate | 132.14 | 2 | 1.319 | 13.192 |
| Urea | 60.06 | 2 | 0.560 | 5.996 |
| Sodium nitrate | 84.99 | 1 | 1.697 | 16.969 |
| Arginin | 174.2 | 4 | 0.870 | 8.695 |
| Cystein/HCl | 157.62 | 1 | 3.147 | 31.471 |
| Ammonium nitrate | 80.04 | 2 | 0.799 | 7.991 |
| Glycin | 75.07 | 1 | 1.499 | 14.989 |

Table 2: Residual glucose and produced hydrogen after growth of N. cameroonii in dependence from the amount of hydrogen added pre inoculation and the pressure inside the bottle at the beginning of the experiment. All values are mean values of a triplicate with standard deviation.

| initially added hydrogen [ml] | initially added hydrogen [mmol] | initial pressure  [bar] | residual glucose  [mmol] | produced hydrogen  [mmol] |
| --- | --- | --- | --- | --- |
| 0 | 0.000±0.000 | 1.227±0.009 | 0.291±0.030 | 0.221±0.016 |
| 2.5 | 0.093±0.001 | 1.207±0.012 | 0.081±0.047 | 0.222±0.032 |
| 5 | 0.188±0.000 | 1.213±0.059 | 0.123±0.050 | 0.191±0.027 |
| 7.5 | 0.277±0.005 | 1.293±0.017 | 0.188±0.106 | 0.201±0.015 |
| 10 | 0.381±0.005 | 1.310±0.024 | 0.076±0.060 | 0.170±0.023 |
| 12.5 | 0.458±0.004 | 1.360±0.008 | 0.081±0.040 | 0.169±0.029 |
| 15 | 0.565±0.007 | 1.387±0.031 | 0.050±0.065 | 0.136±0.034 |
| 20 | 0.736±0.009 | 1.453±0.033 | 0.026±0.011 | 0.112±0.020 |

Table 3: Output from the multiple linear regression in Origin evaluating the effects of the parameters hydrogen start (mmol) and pressure start (bar) on the dependent variable produced hydrogen (mmol).

| Number of Points | 48 |  |  |  |
| --- | --- | --- | --- | --- |
| Degrees of Freedom | 45 |  |  |  |
| Residual Sum of Squares | 0.02027 |  |  |  |
| R-Square (COD) | 0.73165 |  |  |  |
| Adj. R-Square | 0.71973 |  |  |  |
|  | |  |  |  |
|  | Value | Standard Error | t-Value | Prob>\|t\| |
| Intercept | 0.29548 | 0.01371 | 21.55487 | 2.37948E-25 |
| Pressure start | -0.05299 | 0.00836 | -6.34155 | 9.70107E-8 |
| Hydrogen start | -0.1415 | 0.01354 | -10.45109 | 1.28198E-13 |

*Table 4: Final pressure and produced hydrogen during growth on different N-sources with 0.5 mM Na2S as reducing agent. For Ammonium sulfate* 1 g/l cysteine was used as reducing agent. All values are mean values of a triplicate with standard deviation.*

| N-source | Pressure [bar] | Produced hydrogen [mmol] |
| --- | --- | --- |
| Glutamine | 1.857±0.164 | 0.261±0.049 |
| Ammonium sulfate | 1.790±0.216 | 0.203±0.053 |
| Urea | 1.163±0.009 | 0.002±0.002 |
| Sodium nitrate | 1.163±0.005 | 0.000±0.000 |
| Arginin | 1.170±0.008 | 0.000±0.000 |
| Cysteine | 1.167±0.012 | 0.000±0.000 |
| Ammonium nitrate | 1.603±0.287 | 0.169±0.088 |
| Glycine | 1.150±0.014 | 0.002±0.002 |
| Straw | 1.157±0.005 | 0.000±0.000 |
| Water | 1.200±0.078 | 0.000±0.000 |
| Ammonium sulfate* | 2.053±0.009 | 0.273±0.006 |

# Figures


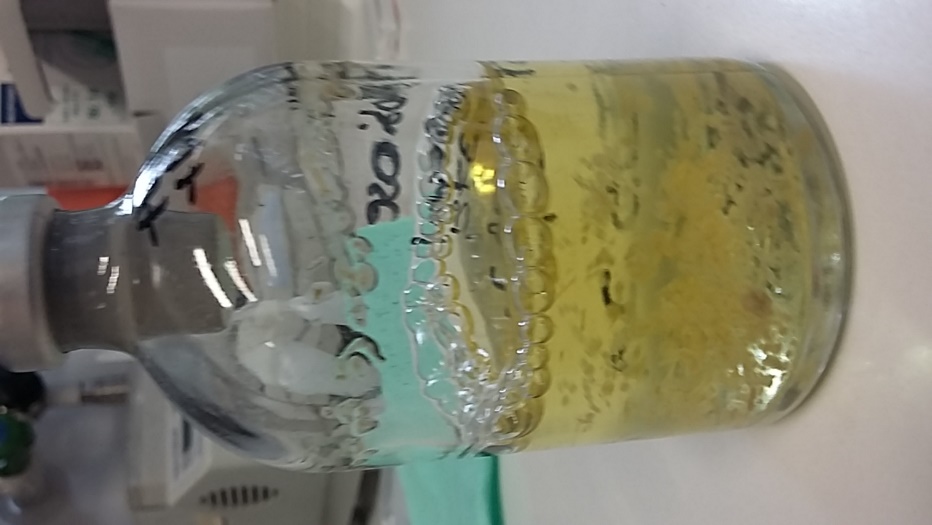


Figure 1: Morphology of Neocallimastix cameroonii during growth on cellobiose while agitated.


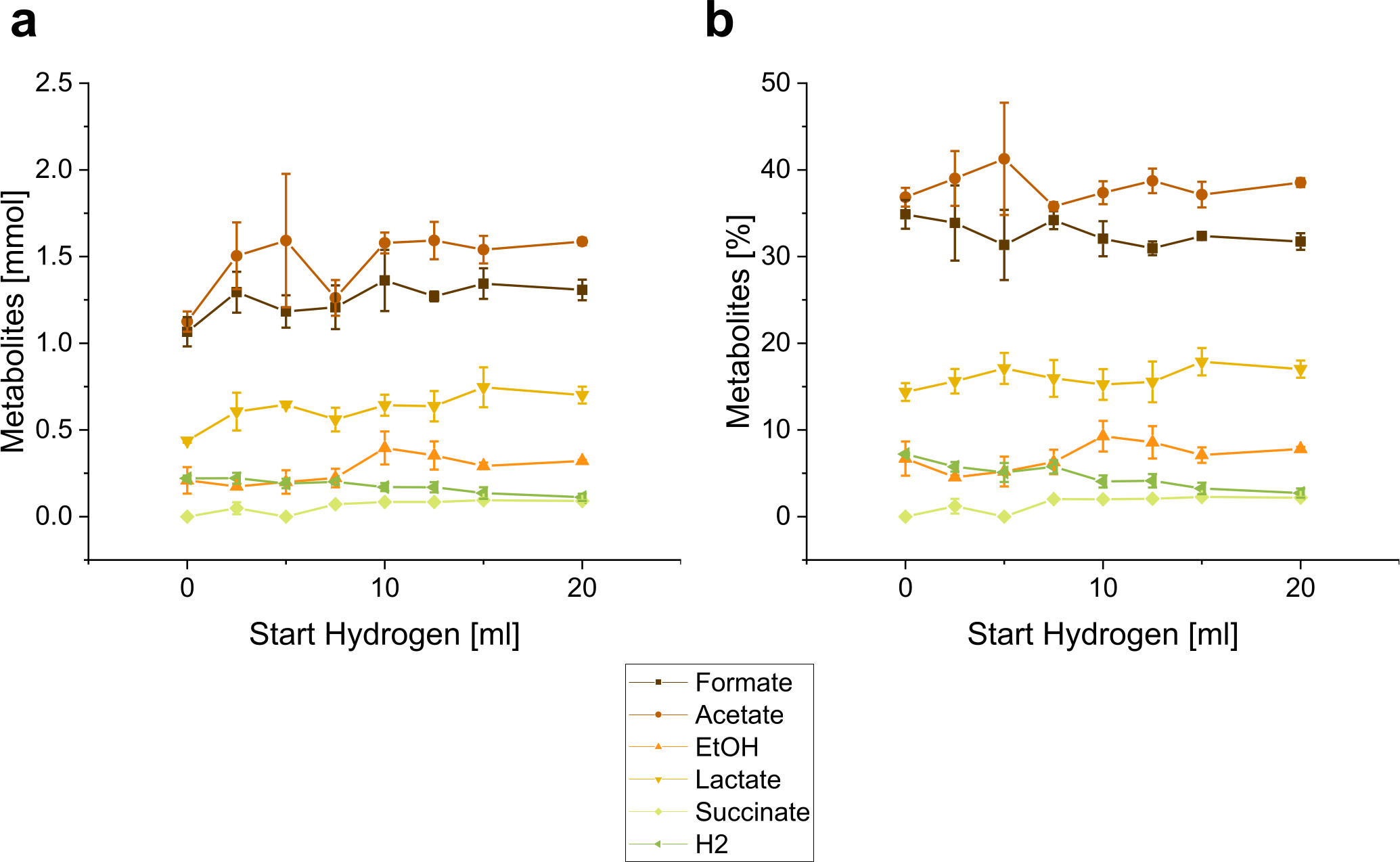


Figure 2: Effect of hydrogen addition on the metabolism of N. cameroonii. a: absolute amounts of the produced metabolites; b: relative amount of the produced metabolites in relation to the total amount of produced metabolites.


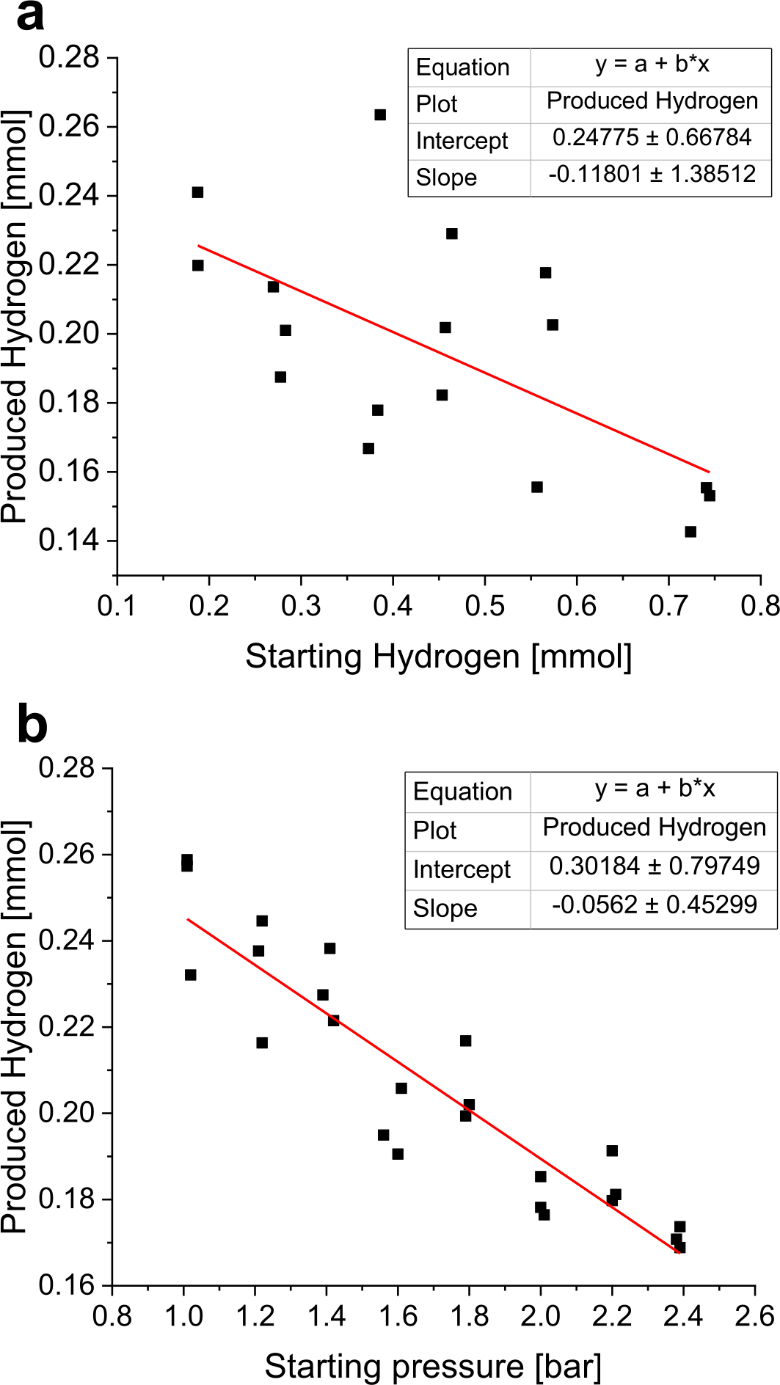


Figure 3: Effect of initial hydrogen and pressure on hydrogen production of N. cameroonii. a: Effect of initial hydrogen; b: effect of initial pressure. Linear fitting was performed with Origin (OriginLabs).
